# New sedimentary evidence reveals a unique history of C_4_ biomass in continental East Asia since the early Miocene

**DOI:** 10.1038/s41598-017-00285-7

**Published:** 2017-03-13

**Authors:** Bin Zhou, Michael Bird, Hongbo Zheng, Enlou Zhang, Christopher M. Wurster, Luhua Xie, David Taylor

**Affiliations:** 1 Key Laboratory of Surficial Geochemistry (Ministry of Education), School of Earth Sciences and Engineering, Nanjing University, Nanjing, China; 2 College of Science and engineering and Centre for Tropical environmental and Sustainability Science, James Cook University, Cairns, Australia; 3 School of Resource, Environment and Earth Science, Yunnan University, Chenggong District, Kunming, China; 4 State Key Laboratory of Lake Science and Environment, Nanjing Institute of Geography and limnology, Chinese Academy of Sciences, Nanjing, China; 5 CAS Key Laboratory of Marginal Sea Geology, Guangzhou Institute of Geochemistry, Chinese Academy of Sciences, Guangzhou, China; 6 Department of Geography, National University of Singapore, Singapore, Singapore

## Abstract

Pyrogenic carbon (PyC) and *n*-alkane data from sediments in the northern South China Sea reveal variations in material from C_4_ plants in East Asia over the last ~19 Ma. These data indicate the likely presence of C_4_ taxa during the earliest part of the record analysed, with C_4_ species also prominent during the mid and late Miocene and especially the mid Quaternary. Notably the two records diverge after the mid Quaternary, when PyC data indicate a reduced contribution of C_4_ taxa to biomass burning, whereas plant-derived *n*-alkanes indicate a greater abundance of C_4_ plants. This divergence likely reflects differences in the predominant source areas of organic materials accumulating at the coring site, with PyC representing a larger source area that includes material transported in the atmosphere from more temperate (relatively cooler and drier) parts of East Asia. Variations in the relative abundances of C_3_ and C_4_ taxa appear to be linked to a combination of environmental factors that have varied temporally and geographically and that are unique to East Asia. A major expansion of C_4_ biomass in warmer subtropical parts of eastern Asia from ~1 Ma and particularly from ~0.4 Ma is later than other parts of the world.

## Introduction

The Calvin-Benson cycle, the process through which plants convert inorganic carbon (C) and water to three C (C_3_) sugar molecules, originated when atmospheric composition was very different from present^1, 2^. One modification to the cycle, leading to reduced photorespiration effects and improved photosynthetic efficiency under certain conditions (e.g. moisture stress and relatively low *p*CO_2_), involves production of four C (C_4_) oxaloacetate as the first-formed product of photosynthesis. Uncertainty surrounds the exact date of origin of this modification. That said, there is no widely accepted evidence of C_4_ taxa pre-dating the Oligocene^3^, with molecular studies indicating that the C_4_ photosynthetic pathway first appeared between 35 and 30 Ma^4^, a period that includes a major decline in *p*CO_2_^5^. Only ~3% of angiosperm species currently support the C_4_ pathway. However, despite being utilized by a relatively small proportion of the global flora, C_4_ taxa – predominantly in the form of grasses – account for a substantial proportion of vegetation cover of Earth^6^, and include important food staples.

An expansion of plants utilising the C_4_ photosynthetic pathway (e.g., tropical grasses) relative to those using the C_3_ pathway (e.g., trees, shrubs and temperate grasses) in the late Miocene constitutes one of the most important biogeographical transformations of the Cenozoic^7–10^. The timing of this expansion appears to have varied geographically, occurring later in East Asia than other parts of the world^11–15^. As evidence has accumulated, however, the expansion of C_4_ plants in East Asia appears to have been phased, rather than a single event, and may have occurred much earlier in some parts of the region^16, 17^, than in others^18^. Determining when and where the C_4_ photosynthetic pathway first appeared on the Asian continent is difficult because direct evidence, in the form of plant remains such as pollen and phytoliths, is rare. Furthermore, when records are available, they are often incomplete; C_4_ taxa often occupy environments that are not conducive to the preservation of organic material *in situ*, and achieving a high level of certainty in distinguishing between many C_4_ and C_3_ taxa in pollen and phytolith records can be problematic.

Stable C isotope (δ^13^C) values for C_3_ plants globally range between −37‰ and −20‰ (average −28.5‰)^19^, while the δ^13^C values for C_4_ plants range from −15‰ to −9‰ (average −13‰)^20–22^. Pyrogenic carbon (PyC), also known as elemental carbon (EC) or black carbon (BC), is produced through the incomplete combustion of biomass and can preserve the original C isotope value of the parent plants^23^. PyC is generally resistant to decomposition, and upon extraction from sediments can be used not only to document the frequency and intensity of past fire activity, but also changes in the relative contributions of C_4_ and C_3_ plants in combusted biomass^15, 16, 23–25^. Similarly, long chain *n*-alkanes (≥*n-*C_27_), which are important building blocks of lipids forming the protective waxy cuticle covering leaf and stem tissue, are resistant to diagenetic alteration^26^. Where herbaceous vegetation predominates, *n*-alkanes of C_31_ and C_33_ are abundant components of sediments, while larger proportions of *n*-alkanes of C_27_ and C_29_ are generally indicative of wooded and forested landscapes (i.e. C_3_ taxa)^27–29^. Thus ratios of measured levels of *n*-alkanes, such as C_31_/C_27_, can be used to reconstruct the relative contributions of herbaceous and woody plants to organic C preserved in sediments^27^. Furthermore, although long chain *n*-alkanes typically have lower δ^13^C values than bulk tissue^30^, fractionation effects can be removed, so that the PyC and long chain *n*-alkanes composition of organic material in sediments can be used as a basis for inferring past variations in the relative abundances of C_3_ and C_4_ plants in, respectively, combusted biomass and vegetation^23, 30, 31^.

The South China Sea (SCS), a marginal sea between Asia and the Pacific Ocean, is dominated climatically by the East Asian monsoon^16, 32, 33^. Sediments accumulating on the bed of the northern SCS, largely supplied by major rivers but also including aeolian material transported by the East Asian Winter Monsoon (EAWM)^34^, provide a potential source of information on environmental variations affecting continental East Asia during the late Cenozoic. To date, there is only one sediment-based record of variations in PyC from the northern SCS^16^. A second core, collected from a deep water site in the southern part of the SCS, has yielded a record of variations in *n*-alkane content of sediment, presumably largely of low latitude origin, for the last 5 Ma^29^. Here we present new data in the form of variations in PyC and *n*-alkanes from the same sediment core from the northern SCS relating to the last ~19 Ma. These new data are compared with published information in order to describe and explain the unique history of C_3_ and C_4_ taxa in East Asia since the early Miocene.

### Study area, materials and methods

**Figure 1:**
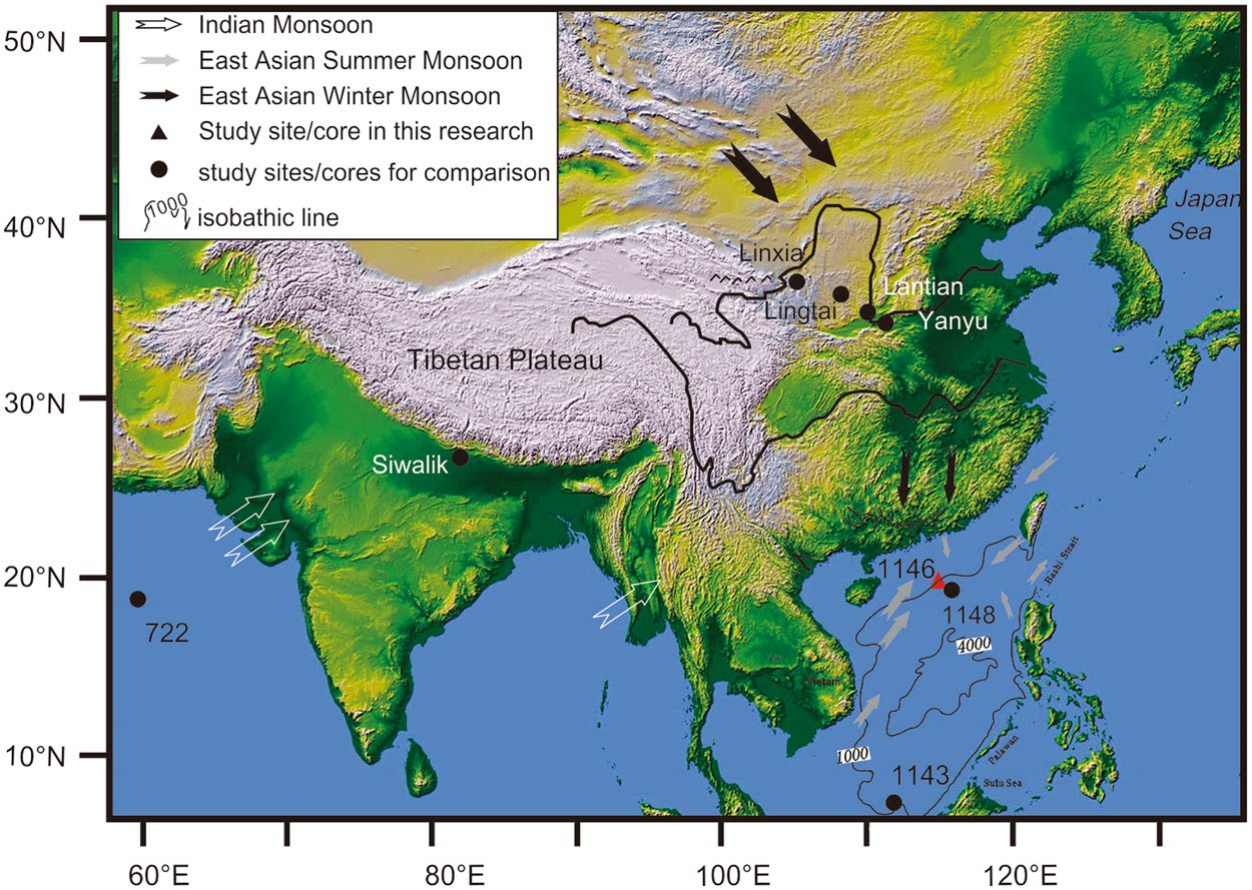
Map showing location of ODP Site 1146 and other important sites mentioned in the paper. The upper left panel contains the legend for the map. Ocean core locations ODP Site 1081 (South Atlantic, 19°37.2′S, 11°19.2′E, 793.8 m water depth), ODP site 717 (Bengal Fan, 0°56′S, 81°23′E, 4734.7 m water depth) and DSDP Leg 10 Site 94 (Gulf of Mexico, 24°20′N, 88°28.2′W, 1793 m water depth) mentioned in the text of the paper are not shown. The map was generated specifically for this paper using the ETOPO5 gridded elevation dataset (Version ETOPO5, http://www.ngdc.noaa.gov/mgg/global/etopo5.HTML) and Coreldraw (Version Coreldraw X7, http://www.corel.com/cn); no copyright problems are envisaged.

The Ocean Drilling Program (ODP) Leg 184, Site 1146 (19°27.4′N, 116°16.4′E, 2092 m water depth) retrieved sediment from a small rift basin on the mid-continental slope of the northern SCS, located less than 50 km to the northeast of ODP Site 1147/1148 (Fig. 1). The sediment sequence obtained at ODP Site 1146 extended to a sub-seafloor depth of 643 m composite depth (mcd). For this study, a total of 149 2-cm-thick sample slices were collected at 4 m intervals between 641 mcd and the surface. Chronological control was established on the basis of palaeomagnetism and biostratigraphy^35^ (S1, Supplementary material), with the ages of sample depths between control points established through linear interpolation (Fig. S1). According to the age-depth model for ODP Site 1146^35^, the sequence of sediments between 641 mcd and the surface covers the last ~19 Ma, with the sampling interval of 4 m adopted in the current research equating to a temporal resolution of ~100 ka.

PyC was extracted using the method of Gustafsson *et al*.^36^. Sediment samples were pretreated with 1 M NaOH, HCl and HNO_3_ to remove inorganic C and some of the organic forms that might lead to BC formation during the pre-combustion step. PyC was then measured following combustion at 375 °C in air for 24 h, with the δ^13^C values for PyC (δ^13^C_PyC_) values determined using a MAT-253 mass spectrometer. Isotopic compositions are expressed as deviations relative to the V-PDB standard with a precision of ±0.2‰ or better. This procedure differs from the oxidation method used to measure PyC (therein termed BC) in sediments from ODP Sites 1147/1148 and the Lingtai Section of the Chinese Loess Plateau (CLP)^16, 37^.

For analysis of high molecular weight *n*-alkanes, samples were freeze-dried before being ground manually. After adding an internal standard (*n*C_24_D_50_), a ~3 g sample was ultrasonically extracted in dichloromethane–methanol (3:1, v/v). Extracts were dried and saponified with 6% KOH–methanol. Hexane was then used to extract hydrocarbons, which were separated by column chromatography into the polar (i.e. alkanol) and non-polar (i.e. alkane) fractions. The δ^13^C values for individual alkanes (δ^13^C_Alk_) were determined using a GV Isochrom II system interfaced to a Hewlett-Packard 5890 gas chromatograph. The gas chromatograph was fitted with a fused Si column HP-5 MS (30 m-0.32 mm-0.25 mm) connected to the combustion interface. Helium was used as the carrier gas with a flow rate of 1.2 mL min^−1^. The temperature was programmed to remain at 80 °C for 2 min, before rising to 220 °C at a rate of 10 °C min^−1^, and then to 290 °C at a rate of 3 °C min^−1^. The temperature was then maintained at 290 °C for 15 min. CO_2_ was used as a reference gas, which was automatically introduced into the isotope ratio mass spectrometer before and after each analysis. The δ^13^C values were calibrated against a standard mixture of *n*-alkanes (*n*C_12_–*n*C_32_) of known isotopic composition (Indiana University, USA) and reported as ‰ relative to Vienna Peedee Belemnite (V-PDB). Replicate analyses showed that the standard deviation for each compound was less than 0.3‰. In order to maintain the reproducibility and accuracy of results, standards were run between samples, and each sediment sample was analyzed at least twice.

The δ^13^C value of atmospheric CO_2_ has not remained constant over time^9, 23, 38, 39^. Moreover, fractionation effects can occur during the production, transport and sedimentation of organic matter^23, 40, 41^, and between atmospheric CO_2_ and plant organic C, as well as varying between different taxa^42, 43^. These sources of variation introduce additional uncertainty into calculations of the relative contribution to combusted biomass and relative abundance in vegetation of C_4_ taxa based on, respectively, δ^13^C_PyC_ and δ^13^C_Alk_, and hence need to be accounted for when interpreting records of ancient δ^13^C. Carbon isotopic enrichment factors for PyC and *n*-alkanes relative to atmospheric CO_2_ (ε_PyC-CO2_ and ε_Alk-CO2_, respectively) were thus applied to calculate C_4_ abundance in the current study (S2 and Fig. S2, Supplementary material).

## Results

**Figure 2:**
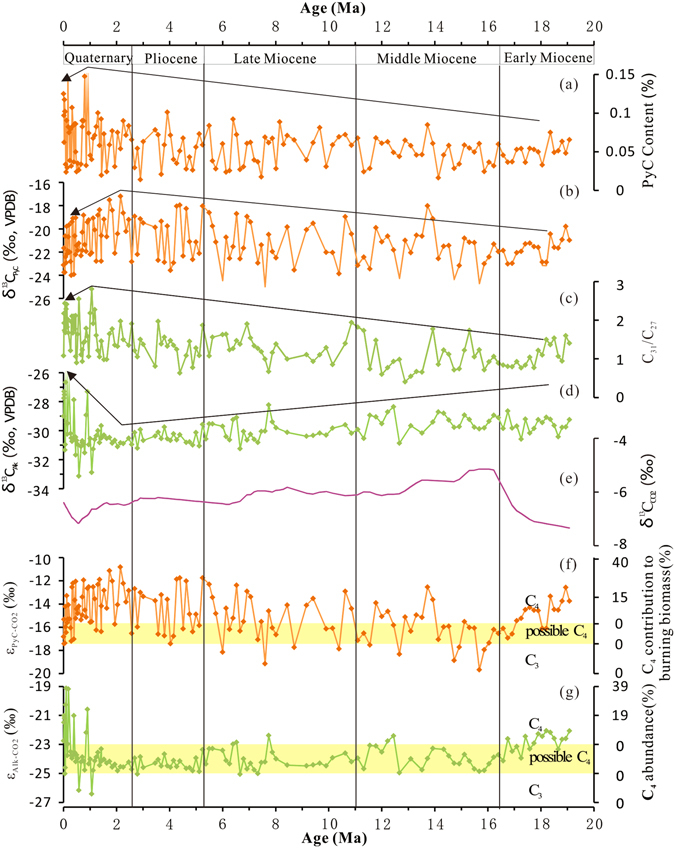
Abundance and carbon isotope composition data from *n*-alkanes and pyrogenic C (PyC) from ODP core 1146: (**a**) PyC content; (**b**) δ^13^C values of PyC (δ^13^C_PyC_); (**c**) C_31_/C_27_ *n*-alkane ratio; (**d**) Average δ^13^C values of long-chain *n*-alkanes (δ^13^C_Alk_); (**e**) Record of δ^13^C of atmospheric CO_2_^39^; (**f**) The contribution of C_4_ taxa to combusted biomass based on carbon isotopic enrichment factor for PyC (ε_PyC-CO2_); (**g**) C_4_ abundance based on carbon isotopic enrichment factor for *n-*alkanes (ε_Alk-CO2)_. Arrows indicate the overall trend in proxies. Two yellow bars show the area of uncertainty in reconstructing the relative prominence of C_3_ and C_4_ taxa based on ε_PyC-CO2_ and ε_Alk-CO2_, and thus the basis for the very conservative estimates of prominence of C_4_ taxa mentioned in the text of this paper.

The PyC content of sediments from ODP Site 1146 (Fig. 2a, Table S1) shows an overall slight increase from the early Miocene through to the Quaternary, ranging from 0.014% to 0.16% (average 0.06%). This trend is superimposed upon strong variability in the data, with the amplitude of variation particularly large from the mid Quaternary. The latter period includes two intervals of high PyC content, centred upon ~1 Ma and 0.2 Ma. δ^13^C_PyC_ values range from −25‰ to −17.2% (average −21.2‰), and also show an overall increase since the early Miocene, before declining after ~2 Ma (Fig. 2b, Table S1). Relatively high (less negative) δ^13^C_PyC_ values date to the early Miocene (~18–19 Ma), mid Miocene (~14 Ma), late Miocene/early Pliocene (~7–4.5 Ma), and to the early to mid Quaternary (~2–1 Ma).

Trends in C_31_/C_27_ ratios (Fig. 2c, Table S1) are broadly similar to those exhibited by PyC; C_31_/C_27_ ratios vary over the last ~19 Ma, showing an overall slight increase to ~1 Ma, with an increased frequency of above average values after ~7.5 Ma, exhibiting greatest variability and highest values from ~1 Ma. In addition, C_31_/C_27_ ratios fluctuate through the early and mid Miocene, with four peaks around ~18 Ma, ~15.5 Ma, ~14 Ma and ~11 Ma.

The weighted mean δ^13^C values of long chain alkanes, determined from (*n*C_27_ * δ^13^C*n*C_27_ + *n*C_29_ * δ^13^C*n*C_29_ + *n*C_31_*δ^13^C*n*C_31_)/(*n*C_27_ + *n*C_29_ + *n*C_31_), is abbreviated to δ^13^C_Alk_. Values of δ^13^C_Alk_ varied from −33.2‰ to −25.6‰ (average −29.9‰) (Fig. 2d, Table S1). Although fluctuating, an overall trend of declining (increasingly negative) values is evident, from the earliest parts of the record (~19 Ma) to the mid Quaternary, with three zones of relatively high (less negative) δ^13^C_Alk_ values dating to around the early Miocene (18–19 Ma), late mid Miocene (11.5–12.5 Ma), and middle and later part of the late Miocene (~7.5–5.2 Ma). δ^13^C_Alk_ values peak (least negative) after ~1 Ma, especially from 0.4 Ma. Over the entire sequence of sediments described here, trends in δ^13^C_Alk_ values are generally opposite to those of δ^13^C_PyC_, particularly from the early Quaternary and especially from ~1 Ma.

Variations in δ^13^C value of atmospheric CO_2_^39^ (Fig. 2e), interpolated to correspond directly with dated δ^13^C_PyC_ and δ^13^C_Alk_ records, were used to derive estimates of ε_PyC-CO2_ and ε_Alk-CO2_, respectively. Conservative estimates of the relative contribution of C_4_ taxa to combusted biomass and vegetation ranged from, respectively, 0–34.7% (average 12.9%) and 0–41.6% (average 9.9%) (Fig. 2f and g). Values of both ε_PyC-CO2_ and ε_Alk-CO2_ indicate the likely presence of C_4_ taxa in East Asia during the early Miocene, later part of mid Miocene and the late Miocene through to the Late Quaternary. Some differences are evident in the two sources of information, however. The ε_PyC-CO2_ data tend to indicate a relatively significant and continuous, though strongly varying, C_4_ contribution to combusted biomass, especially when compared with the corresponding estimated abundance of C_4_ taxa in terrestrial vegetation based on ε_Alk-CO2_ data. The largest differences between the two datasets date to the Pliocene and Quaternary; ε_PyC-CO2_ data indicate an overall substantial contribution of C_4_ grasses to combusted biomass from the late Miocene through to the Quaternary, though with an overall decline evident after ~2 Ma. Lower ε_Alk-CO2_ values indicate a predominance of C_3_ grasses from around the beginning of the Pliocene through to ~1 Ma. This is supported to an extent by the enriched C_31_/C_27_ ratios, which suggest a greater herbaceous contribution to organic C from the late Miocene. An expansion of herbaceous vegetation generally (including a greater proportion of C_4_ grasses at times) from ~1 Ma, particularly from ~0.4 Ma, is suggested by enriched high δ^13^C_Alk_ data values and generally higher average C_31_/C_27_ ratios.

## Discussion

### Deviation of PyC and *n*-alkane records in ODP 1146 from northern SCS

**Figure 3:**
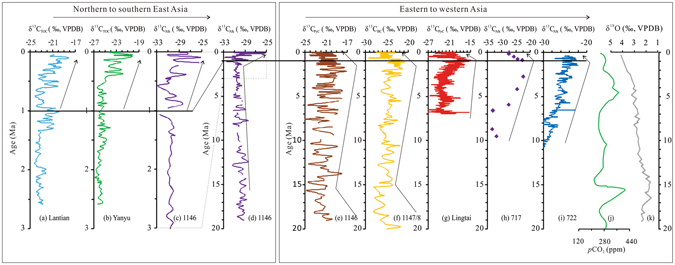
Summary of data for ODP core 1146 compared with similar data from sites proximate to, and on, the continent of Asia. Charts in the left panel summarise data north to south across East Asia. (**a**) and (**b**) δ^13^C_TOC_ from transect sections on Chinese Loess Plateau (from northwest to southeast: Lantian, Yanyu)^12, 18^; (**c**) and (**d**) δ^13^C_alk_ values from core 1146 since the last 3 Ma and since about 19 Ma (this study). The right panel summarises data from a far larger area. (**e**) δ^13^C values of PyC from core 1146 in this study; (**f**) and (**g**) δ^13^C values of black carbon from 1147/8 and δ^13^C values of PyC from Lingtai loess section^16, 37^; (**h**) and (**i**) marine sediments in Indian Ocean (ODP 717)^62^ and Bengal Fan (ODP 722)^63^; (**j**) Published *p*CO_2_ records since the mid-Miocene based on a compilation of CO_2_ curves (green line)^59^; (**k**) Simplified composite δ^18^O record of deep-sea benthic foraminifera as a proxy for global temperature variations^65^. Dashed line shows in enlarged form the record for δ^13^C_alk_ in core 1146 since the last ~3 Ma. Solid line highlights the divergence of δ^13^C values occurring since the mid Quaternary. Arrows show direction of overall change in the data.

Differences in *n*-alkane and PyC data from the same core likely reflect differences in both provenance of the C on which the measurements are based, and in the mode of transport of the C from the terrestrial source to the site of deposition. PyC is formed at high temperatures during the combustion of biomass and emitted to the atmosphere before a proportion is deposited in sedimentary environments^44^, with the amount deposited dependent on atmospheric transport (influenced by wind velocity and direction and topography), precipitation and depositional processes. Several studies have indicated that PyC preserved in deep-sea sediments in the SCS originates via long-range transport from burning in central and southeastern Asia^45–51^ (S3, Supplementary material). Atmospheric transport from these sources is particularly active during EAWM, when dry continental conditions can also enhance the likelihood of vegetation fires^52, 53^. In general, highly seasonal and semi-arid climate conditions will result in biomass that is prone to burning, at least towards the end of the dry season, and, on occasion, in intense fires^24^. Thus levels of PyC accumulating in sediments in the SCS are affected not only by the extent and composition of vegetation available for combustion, but also by burning regime and by variations in the EAWM in particular^24^. Variations in PyC therefore likely represent an integration of conditions over a broad geographic area, with the source area of PyC accumulating in the northern SCS potentially including cold-adapted vegetation in more temperate, higher altitude and latitude parts of continental East Asia^16^. This is evident in similarities between variations in δ^13^C_PyC_ measured at ODP Site 1146 discussed here and δ^13^C_BC_ from Site 1148^16^, and from the CLP over the last ~7 Ma^15^ (Fig. 3).

By comparison, *n*-alkanes are mainly derived from plant leaves^30^. Surface sediment n-C_29_ concentrations are in good agreement with data on pollen from approximately the same suite of modern sediments in the northern SCS^54, 55^. The main delivery mechanism for *n*-alkanes is likely to be dominated by fluvial inputs and then marine currents via the Bashi and Taiwan straits^55^. Some *n*-alkanes may also have been deposited from the atmosphere, along with aeolian dust and aerosols associated with biomass burning^56^. However, the fact that levels of *n*-alkanes and PyC measured in the same sediment samples are not well-correlated suggests the two proxies have different provenances (Fig. S3). Possibly therefore, *n*-alkanes accumulating in the past in sediments in the northern SCS originated from relatively well-vegetated parts of subtropical East Asia, rather than as a result of long distance aeolian input from more temperate land to the north, where the vegetation cover is comparatively sparse^24^. Catchments draining into the northern SCS are likely to be the dominant means by which organic material containing plant lipids is transported mainly from sub-tropical latitudes^55^, with variations in δ^13^C_Alk_ therefore affected by a different set of drivers to PyC abundance, including changes in monsoonal precipitation and fluvial discharge, catchment conditions and the extent and proximity of land relative to ocean^57^.

### Variations in C_4_ contribution to vegetation and combusted biomass in East Asia recorded by PyC and *n*-alkane data

Variations in the prominence of C_4_ taxa estimated from ε_PyC-CO2_ (relative contribution to combusted biomass) and ε_Alk-CO2_ (relative abundance in vegetation) are generally in phase with the δ^13^C_PyC_ and δ^13^C_Alk_ records, respectively. The δ^13^C_PyC_ values are also in general agreement with variations in δ^13^C_BC_ from ODP sites 1147/1148^16^ (Fig. 3), notwithstanding the aforementioned systematic offset in values due to differences in the analytical techniques employed^23^. Variations in δ^13^C_Alk_ are also within a similar range to that observed in a previously reported record from the northern SCS^57^ covering a shorter period of time than that discussed here.

The presence of C_4_ taxa during the early (~18.5 Ma) and mid Miocene (~14 Ma) and from the late Miocene (~7.5 Ma) is supported by existing data^9, 15–17, 58^. Variations in abundances of C_4_ taxa have been attributed to the changes in *p*CO_2_^10, 59^ and to evolution of East Asian monsoon^12, 16^, seasonality^18^, aridity and/or fires^15, 58^. Intensification of the East Asian Summer Monsoon (EASM) is thought to date to about 24 Ma, with a peak in intensity during the mid Miocene, a weakening after ~8 Ma and strengthening again during the late Pliocene/early Quaternary^29, 30^, while strengthening of the EAWM is thought to have occurred 16–14 Ma, ~8 Ma, and ~3 Ma and to have further intensified from the mid Quaternary^60^. Increasing PyC content indicates rising levels of biomass burning, with periods of relatively high contributions to combusted biomass from C_4_ taxa evident ~14 Ma, ~7–4.5 Ma and ~2.2–1.5 Ma. Increased contributions of C_4_ taxa to biomass burning may represent a climatically promoted competitive advantage for C_4_ photosynthesis, due not only to a strengthened EAWM and greater aridity^57, 61^, but also to increased seasonality as a result of renewed intensification of the EASM, especially from ~3 Ma. From the mid Quaternary, both above average PyC levels and C_31_/C_27_ ratios could be a response to increased aridity, possibly due to a strengthening of the EAWM following further expansion of the northern hemisphere ice sheets^12, 29, 32^. The contribution of C_4_ taxa to combusted biomass declines from ~2 Ma, presumably because of a greater presence of C_3_ plants in the source region for PyC. Evidence from the CLP for increased prominence of C_3_ taxa during much of the Quaternary may represent a combination of a more intense EAWM and reduced temperatures during the main growing season in more temperate parts of the region, with both factors favouring C_3_ photosynthesis^15, 62^.

At lower latitudes, C_3_ taxa were prominent from the mid Miocene to the Pliocene/Quaternary boundary, under warm and humid climate conditions presumably owing to the influence of the EASM^33^. Reduced moisture and increased seasonality from the late Pliocene through to ~1 Ma, with the majority of precipitation occurring during the growing season^29^, could have facilitated sustained increases in the relative abundance of C_3_ grasses, as evidenced by relatively lower ε_Alk-CO2_ values and higher C_31_/C_27_ ratios. As seasonal conditions developed and vegetation canopies became more open, expansion of C_4_ biomass could have become tightly coupled in a feedback relationship to changes in burning regime and aridity that effectively selected against shrubs and trees (i.e. C_3_ species) and promoted further expansion of grasslands in which C_4_ taxa were prominent. An expansion of C_4_ taxa in subtropical East Asia from ~1 Ma, and especially ~0.4 Ma, may have been triggered by increased seasonality, aridity and changes to the fire regime.

### A synthesis of evidence for C_4_ variations across Asia

δ^13^C values from fossil enamel and soils from the northwestern CLP (the northeastern margin of the Qinghai–Tibet Plateau) suggest increased abundance of C_4_ taxa from ~3 Ma, with C_4_ taxa becoming a significant component of the local vegetation around ~1.0 Ma^13^. An expansion of C_4_ biomass ~1.0 Ma and, at lower altitude, ~0.4 Ma, on the southeastern parts of the CLP is evident in δ^13^C data from Yanyu (620 m amsl) and Lantian (769 m amsl)^12, 18^ (Fig. 3). The evidence from Lantian and Yanyu is thus consistent with our δ^13^C_Alk_ data from the northern SCS.

The δ^13^C values of *n*-alkanes obtained from palaeosols associated with the Siwalik Group and marine sediments from the Indus (ODP Site 722) and Bengal (ODP Site 717C) fans^63, 64^, dating to 12–11 Ma, indicate a predominantly C_3_ flora during the mid Miocene (Fig. 3). A subsequent expansion of C_4_ taxa during the late Miocene in western and southern Asia has been linked to aridification^64^, although other factors, such as increased burning^15, 25^ and lower *p*CO_2_ may have played a role. Evidence of an expansion of C_4_ biomass from ~8–6 Ma that peaked in the early to mid Quaternary in Asia, the South Atlantic (ODP Site 1081)^65^, and Gulf of Mexico (DSDP Site 94)^9^ implies the possible effect of global decreases in moisture availability along with ice sheet formation and expansion that commenced during the late Miocene, as recorded in δ^18^O variations preserved in the tests of foraminifera^66^.

The detailed reconstruction of variations in the prominence of C_4_ taxa estimated from ε_PyC-CO2_ and ε_Alk-CO2_ is limited by a number of uncertainties, including the effects of changes in atmospheric *p*CO_2_ and fractionation due to moisture stress^42^, and potentially also O_2_/CO_2_ ratios^67^. Notwithstanding these uncertainties, the available evidence is broadly consistent with the conclusion that the rise to prominence of C_4_ taxa over a substantial proportion of subtropical East Asia differed in timing, likely reflecting local differences in the relative strengths of the suite of potential causal factors. Beginning in the late Pliocene, increased seasonality – with the majority of precipitation occurring during the growing season – and aridity overall could have facilitated sustained increases in the relative abundance of open vegetation in which C_4_ taxa were important components. As seasonal climate conditions developed and vegetation canopies became more open, expansion of C_4_ biomass may have become tightly coupled in a feedback relationship with changes in burning regime that effectively selected against shrubs and trees (i.e., C_3_ species) and promoted further expansions of C_4_ grasslands^15, 64, 68^. Lower temperatures associated with continued uplift of central Asia coupled with a strengthened EAWM and the effects of fluctuating Quaternary ice sheets after ~2 Ma could have shifted habitats previously characterized by a relatively high proportion of C_4_ taxa back within an envelope of environmental conditions that were more suited to C_3_ species. From around the same time, but at lower altitudinal and subtropical parts of the region, aridity, enhanced by an expanded coverage of ice in the NH and large-scale hydrological dynamics, may have forced an abrupt expansion of C_4_ biomass^29^, thereby contributing to a history of C_3_/C_4_ variations in East Asia that is unique for the continent and more widely.

## Conclusions

The abundance and C isotope composition of *n*-alkanes and PyC in samples from a sediment core from the northern SCS are used to reconstruct a history of variations in the relative prominence of C_4_ and C_3_ taxa in East Asia over the last ~19 Ma. Although present throughout the record, C_4_ taxa appear to have been prominent at times during the Miocene and especially from the late Pliocene to mid Quaternary. However, *n*-alkane (a proxy of relative contribution to vegetation) and PyC (a proxy of relative contribution to combusted biomass) isotope data diverge from the mid Quaternary, most likely reflecting differences in provenance, with PyC representing a larger source area that includes higher altitude and more temperate parts of the region, and atmospheric processes as the primary means of transporting organic material from the interior of the continent to the coring site. Temperatures in these already cooler parts of the continent appear to have fallen to levels too low to support C_4_ taxa from the mid Quaternary. New evidence presented in this paper in the context of existing data indicates that variations in the level and seasonality of rainfall and associated changes in biomass burning appear to have been important drivers of changes in the relative contributions of C_3_ and C_4_ taxa to East Asian vegetation throughout much of the past ~19 Ma. An expansion of C_4_ taxa at low altitude/latitude from ~1 Ma, and especially ~0.4 Ma, may have been triggered by increased aridity and changes to the fire regime. This expansion commenced more recently than in other parts of the tropics and sub-tropics, and is one characteristic of a novel history of variations in C_3_ and C_4_ taxa in continental East Asia.

## Electronic supplementary material

Supplementary material
