# Supplementary material for: New sedimentary evidence reveals a unique history of C4 biomass in continental East Asia since the early Miocene
Source: Sci Rep. 2017 Mar 13;7:170. doi: 10.1038/s41598-017-00285-7 (PMC5428023; doi:10.1038/s41598-017-00285-7)
Supplement: Supplementary file 1 — Supplementary material [file 41598_2017_285_MOESM1_ESM.doc]

**Supplementary material**

**New sedimentary evidence reveals a unique history of C_4_ biomass in continental East Asia since the early Miocene**

Bin Zhou^a, *^, Michael Bird^b^, Hongbo Zheng^c^, Enlou Zhang^d^, Christopher M. Wuster^b^, Luhua Xie^e^ and David Taylor^f^

^a^*Key Laboratory of Surficial Geochemistry (Ministry of Education),* *School of Earth Sciences and Engineering, Nanjing University, Nanjing, China*

^b^*College of Science and engineering and Centre for Tropical environmental and Sustainability Science, James Cook University, Cairns, Australia*

^c^*School of Resource, Environment and Earth Science, Yunnan University, Chenggong District, Kunming, China*

^d^*State Key Laboratory of Lake Science and Environment, Nanjing Institute of Geography and limnology, Chinese Academy of Sciences, Nanjing, China*

^e^*CAS Key Laboratory of Marginal Sea Geology, Guangzhou Institute of Geochemistry, Chinese Academy of Sciences, Guangzhou, China*

^f^*Department of Geography,* *National University of Singapore, Singapore*

***Corresponding author**: School of Earth Sciences and Engineering, Nanjing University, 163 Xianlin Ave., Nanjing 210023, China. Tel: +86 25 89687522; Fax: +86 25 89687522. Email: [zhoubinok@nju.edu.cn](mailto:zhoubinok@nju.edu.cn)

**Supplementary S1. The chronostratigraphic framework:**

The chronostratigraphic framework for ODP Site 1146 was established on the basis of the lithostratigraphy, magnetostratigraphy and biostratigraphy^1^ with the estimated age of individual sample depths based on linear interpolation between control points. The sequence of sediments extended to 640 mcd, covering the last ~19 Ma. Three lithologic units (I, II and III) were identified at Site 1146. Unit I consists of late Pliocene and Quaternary-age nannofossil clay extending from 0 to 242.68 mcd (Fig. S1). Unit II comprises mid Miocene to late Pliocene foraminifera and nannofossil clay from 242.68 to 553.02 mcd, while Unit III is made up of early to mid Miocene nannofossil clay from 553.02 to the base at 642.31 mcd. The Pliocene/Quaternary boundary is constrained by the first occurrence (FO) of medium-sized *Gephyrocapsa* spp. and the last occurrence (LO) of *Globigerinoides fistulosus* and *Discoaster brouweri*, and is located between 185.4 and 195.1 mcd. The Miocene/Pliocene boundary is constrained by the LO of *Triquetrorhabdulus rugosus* and the LO of *Discoaster quinqueramus*, between 308.4 and 317.9 mcd, respectively. The Neogene section at Site 1146 yielded 32 calcareous nannofossil events, 27 planktonic and one benthic foraminiferal event, and three paleomagnetic datum levels.


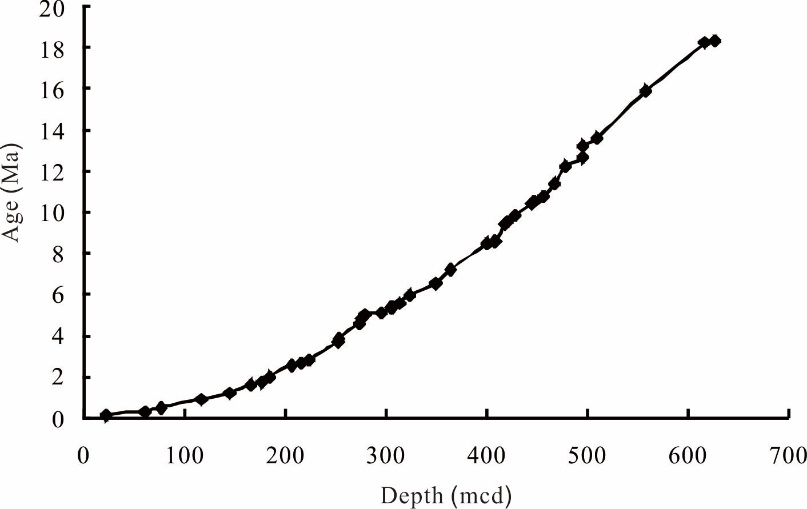


Fig. S1: Age-depth model of ODP core 1146^1^. The base of the sequence of sediments with composite depth of 641 mcd is estimated at ~19 Ma.

**Supplementary S2. C_4_ plant abundance calculation:**

The stable C isotope (δ^13^C) values for C_3_ and C_4_ plants range, respectively, from between -32‰ and -20‰ (average -27‰) and -15‰ to -9‰ (average -13‰)^2^. These average values are slightly different from those in references^3,4^: -28.5‰ (C_3_) and -12.5‰ (C_4_). The stable C isotope composition of organic matter in soils on the Chinese Loess Plateau (CLP) suggest that the δ^13^C values of modern C_3_ and C_4_ plants are vary slightly when compared with the above average values, although well within the stated ranges, − 27.3‰ for C_3_ plants^5,6^, and −12.6‰ for C_4_ plants^5^. In the current research, we use mean δ^13^C values for C_3_ and C_4_ plants of -27 ‰ and -13‰ as end-member values, assuming carbon isotope composition of +1 ‰ enrichment for PyC compared with that of the original source plants^7,8^, and possible maximum +1‰ fractionation for PyC during the pretreatment method of CTO-375 ^9^. In addition, we adopted a very conservative approach to reconstructing the proportional representation of C_4_ taxa in combusted biomass. This involved adding the 1σ value of ca. 2‰ to the average end-member values in order to account for possible moisture stress effects on C_3_ taxa^4,10^. Assuming the average contemporary δ^13^C_CO2_ value is -7.8‰^11,12^, we derived epsilon (ε) end-member values for _PyC-CO2_ (ε_PyC-CO2_) through equation (1) for C_3_ and C_4_ plants of -15.3‰ and -2.2‰, respectively.

ε_PyC-CO2_ =（δ^13^C_PyC_-δ^13^C_CO2_）/（δ^13^C_CO2_+1000）*1000 (1)

ε_PyC-CO2_ =ε_C3PyC-CO2_*f_C3-PyC_ +ε_C4PyC-CO2_*f_C4-PyC_ (2)

f_C3-PyC_ +f_C4-PyC_=1 (3)

In equation 1, δ^13^C_PyC_ and δ^13^C_CO2_ represent the carbon isotopic composition of PyC and atmospheric CO_2_, respectively. Estimations of differences (fractionation) between δ^13^C_PyC_ and δ^13^C_CO2_ (ε_PyC-CO2_) over the last ~19 Ma were obtained from our determinations of δ^13^C_PyC_ and δ^13^C_CO2_ variations via equation (1), as shown in Fig. S2d, using two methods.

In the first method, we used published δ^13^C_CO2_ values derived from benthic foraminiferal records (the black line on Fig S2b)^13^. Applying the ε_PyC-CO2_ values and end-member values for C_3_ and C_4_ plants, we estimated the fractions of each photosynthetic type via equations (2) and (3). The C_4_ abundances recovered ranged from 0 to 34.7%, with an average value of 13.2% (the black line on Fig S2e).

Carbon isotope discrimination by C_3_ plants is also sensitive to atmospheric *p*CO_2_^14,15^). In order to calibrate the carbon isotopic discrimination of C_3_ plants due to secular changes of *p*CO_2_, we used the atmospheric CO_2_ concentration curve^16^ to calculate variations over time in the discrimination value of C_3_ end-members [Δ(Δ^13^C)], and epsilon values for the C_3_ end-member (ε_C3-CO2_) (Fig. S2b), according to the method of Schubert and Jarhen^14,15^. First, we used equation (4) to calculate S^15^, the ‰ change in discrimination per ppm change in CO_2_.

S=(0.21) (28.26)2/[28.26+0.21(*p*CO_2_+25)]^2^ (4)

The change in discrimination of C_3_ plants that results from a change in *p*CO2 [Δ(Δ^13^C)] can then be described by equation (5), following the method of Schubert & Jaheren^15^ and based on the variations in *p*CO_2_ concentration from Beerling & Royer^16^ .

Δ(Δ^13^C)= [(28.26)(0.21)(*p*CO_2(t)_+25] / [28.26+0.21(*p*CO_2(t)_+25)] – [(28.26)(0.21) (*p*CO_2(t=0)_+25)] / [(28.26)+(0.21) (*p*CO_2(t=0)_+25)] (5)

Δ(Δ^13^C)= Δ^13^C (t)- Δ^13^C (t=0) (6)

Δ^13^C (t)= (δ^13^C_CO2_–δ^13^C) / (1 +δ^13^C/1000) (7)

The average Holocene δ^13^C_CO2_ value of -6.4‰ was taken as a reference for when *p*CO_2_ is 270 ppm, thus yielding a Δ^13^C (t=0) value of the C_3_ end-member based on equation (7). We then estimated the discrimination for the C_3_ end-member through the last 19 Ma (Δ^13^C(t)) based on equation (6). We obtained the δ^13^C value of the C_3_ end-member by using the values for the δ^13^C value of atmospheric CO_2_ from Tipple el al.^13^ through equation (7). Then we calculated epsilon values for both the C_3_ endmember (ε_C3PyC-CO2_) and for the observed values by using Tipple et al.^13^ for δ^13^C values of atmospheric CO_2_ (ε_PyC-CO2_) through equation (1), these being shown in Fig. S2c (purple line) and Fig. S2d, respectively, and then determined C_4_ contribution to the total vegetation or to the combusted biomass according to equations (2) and (3). The estimated contribution of C_4_ taxa to combusted biomass based on this method ranged from 0-35.8% (green line in Fig S2e).

We applied similar calculations for recovering C_4_ abundance in vegetation from *n*-alkane data. In the first method, we chose a C_3_ *n*-alkane mean value of -33.1‰ and a C_4_ n-alkane mean value of -21.7‰ as two-end members of modern plants^11,12^, but used a 1σ value of ca. +2 ‰ higher than average end-members in order to account for factors such as water stress effects. Assuming a contemporary average δ^13^C_CO2_ value of -7.8‰^12^, we derived ε_Alk-CO2_ end-members through equation (9) for C_3_ and C_4_ plants of -23.5‰ and -12‰, respectively.

ε_Alk-CO2_ =（δ^13^C_Alk_-δ^13^C_CO2_）/（δ^13^C_CO2_+1000）*1000 (9)

ε_Alk-CO2_ =ε_C3Alk-CO2_*f_C3-alk_ +ε_C4alk-CO2_*f_C4-alk_ (10)

f_C3-alk_ +f_C4-alk_=1 (11)

In equation 9, δ^13^C_Alk_ and δ^13^C_CO2_ represent the δ^13^C value of *n*-alkane and atmospheric CO_2_, respectively. Estimations of ε_Alk-CO2_ over the last ~19 Ma were obtained from our determinations of **δ**^13^C_Alk_ and δ^13^C_CO2_ variations through equation (9) (Fig. S2f). In the first method, the δ^13^C_CO2_ variations are from published data based on benthic foraminiferal records (Fig S2b black line) ^13^. Applying the ε_Alk-CO2_ variation values and ε_Alk-CO2_ end-member values for C_3_ and C_4_ plants enabled the recovery of the proportional representation of material from C_3_ and C_4_ taxa through equations (10) and (11). The estimated contribution of C_4_ taxa to vegetation thus ranged from 0-38.6% (the black line in Fig S2g).

In the second method, we used the atmospheric CO_2_ concentration curve^16^ (Fig S2a green line) in accordance with the method Schubert and Jarhen^14,15^ to obtain the change in discrimination [Δ(Δ^13^C)] that results from a change in *p*CO_2_ in order to determine epsilon values for both the C_3_ end-member (ε_C3Alk-CO2_) and for the observed values ε_Alk-CO2_ (similar method with ε_PyC-CO2_), these being shown in Fig. S2c (blue line) and Fig. S2f, respectively. C_4_ abundance obtained by this method ranged from 0-37.7 % (the green line in Fig S2g).

The calculated C_4_ contributions to combusted biomass and terrestrial vegetation established through the two different methods are similar. In the main text, we refer to the results from the first method.


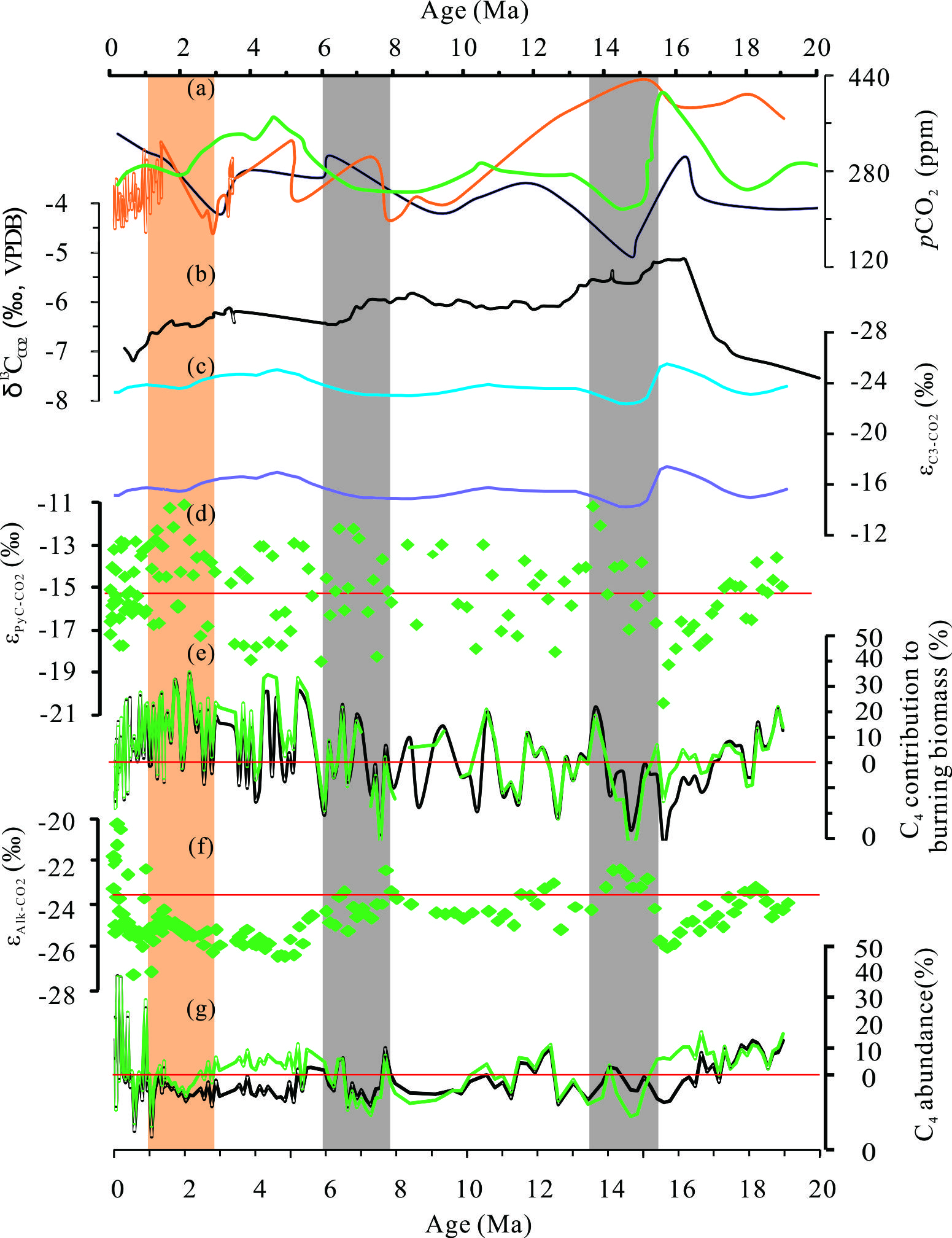


Fig S2: (a) Published *p*CO_2_ records since the mid-Miocene using different methods. Foram δ^11^B-derived *p*CO_2_ is from reference^17^ (black line), and B/Ca ratio-derived *p*CO_2_ is from reference^18^ (orange line), and compilation of variations in CO_2_ concentration data^16^ (green line); (b) The δ^13^C value of atmospheric CO_2_ (δ^13^C_CO2_) over the last ~19 Ma from reference^13^ (black line). (c) epsilon values of C_3_ endmember for PyC and alkanes from core 1146 over the last ~19 Ma (ε_C3PyC-CO2_ and ε_C3Alk-CO2_), with ε_C3PyC-CO2_ in purple and ε_C3PyC-CO2_ in blue.; (d) ε_PyC-CO2_ values from core 1146 over the last ~19 Ma; (e) Comparison of two methods used to estimate the proportional contribution of C_4_ taxa to combusted biomass based on PyC data (results obtained from the first method referred to in the text in S2 are shown as a black line, those from the second method as a green line); (f) ε_Alk-CO2_ values from core 1146 over the last ~19 Ma; (g) Comparison of two methods used to estimate the relative abundance of C_4_ plants in vegetation based on n-alkane data in this study (results based on the first method are shown as a black line, those from the second method as a green line).

**Supplementary S3. Provenance of PyC and n-alkanes in northern SCS:**

PyC, measured by the CTO-375 method^19^, usually comprises highly condensed, fine particles. Pyrogenic carbon formed at high temperature is emitted to the atmosphere and then, depending on atmospheric transport, wash-out and depositional processes, a proportion may be trapped in sedimentary environments^20^. Observational and modern environmental surveys suggest that PyC accumulating in the deep SCS is significantly influenced by long-range atmospheric transport from Asia, mostly through a strong East Asia Winter Monsoon (EAWM)^21^. East-southern and central Asia are regarded as the most significant source regions for PyC^22,23^. Lin et al.^24^ analyzed satellite data and found that aerosol mixed with aeolian dust from China is transported to the northern SCS in winter.

Backward trajectory analysis shows that the air masses arriving at the northern SCS mainly originated from the Asian continent^25^. *In situ* measurements provide strong evidence that aeolian dust from Asia can reach the northern SCS as a result of long-range transport^26^. In addition to the near-surface aerosol transport, a significant upper layer (3-4 km) transport of material from biomass burning was observed^27^. These results suggest that emissions from both continental China and Southeast Asia could have a significant impact on the aerosol loading and other aerosol properties over the SCS, including PyC, with transport greatest during periods of intensified EAWM activity. Grain size records of surface and core samples from northern SCS indicate that variations in the intensity of wet summer and dry winter monsoon control the fluvial and/or aeolian sediment supply to this area^28^. During the strong EAWM, for example during glacial periods, sediments accumulating at the site for core 17940 (adjacent to our core 1146) are free of a fluvially-derived component and dominated by aeolian-dust^28^.

By contrast, fluvial runoff is responsible for the transport of terrestrial material from southern China, particularly in association with a strong East Asia Summer Monsoon^28^. Surface sediment data from the northern SCS suggest a transportation route via the Bashi and Taiwan straits^29,30^. Certainly, *n*-alkanes can also be transported along with aeolian dust and along with other material associated with biomass-burning. For example, biomass burning in the southern/southeastern China via long-range transport was suggested to be a major source for nonpolar organic compounds (NPOCs) in aerosols over the north SCS^31^. However, *n*-alkanes are mainly derived from the waxy protective cuticles on plant leaves, and it is likely that *n*-alkanes accumulating in the past in sediments in the north SCS largely originated from relatively well-vegetated parts of subtropical, southern China. Moreover, sedimentary indicators of biomass burning from the CLP^32^ and northern SCS^33^ show a high level of consistency (Fig. S3a), while PyC and total *n*-alkanes from the same core show no good correlation (Fig. S3b). Based on this analysis, and our data, we conclude that PyC likely represents an integration of conditions over a broad geographic area, with burning regime and intensity of the EAWM in particular potentially powerful drivers of variations in C_3_:C_4_ ratios, while *n*-alkanes are mainly derived from subtropical regions bordering the SCS.


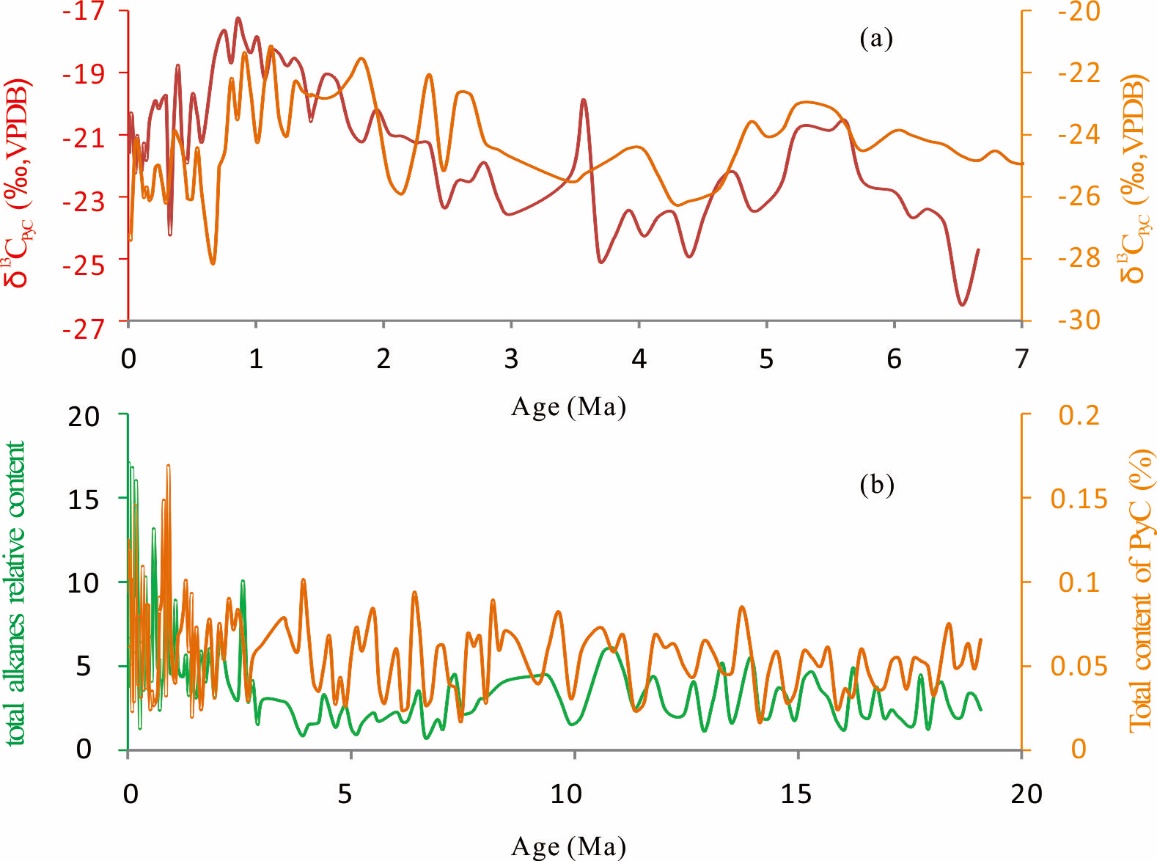


Fig. S3: (a) PyC record from Chinese Loess Plateau (CLP) (red line)^32^; and northern SCS (orange line)^33^ extracted and measured in chemical oxidation method reveal a high level of consistency, indicating a climatic and/or provenance correlation. (b) PyC content (orange line) and total *n*-alkanes are not well correlated, with alkanes having increased largely since the late Pliocene (ca. 3 Ma). This is consistent with increased input of material to the northern SCS via the Taiwan Strait^34,35^.

**Table S1**: Summary of organic carbon data from sediment core material collected from ODP Site 1146

| **mcd（m）** | **Age (Ma)** | **PyC%** | **δ^13^C_pyc_(‰)** | **C_31_/C_27_** | **δ^13^C_Alk_ (‰)** |
| --- | --- | --- | --- | --- | --- |
| 0.49 | 0.009 | 0.12 | -23.11 | 1.08 | -29.01 |
| 4.71 | 0.018 | 0.10 | -21.65 | 2.17 | -27.77 |
| 8.95 | 0.029 | 0.06 | -23.74 | 1.93 | -27.32 |
| 13.20 | 0.041 | 0.12 |  | 1.74 | -27.52 |
| 17.45 | 0.054 | 0.10 | -23.74 | 2.43 | -31.35 |
| 21.70 | 0.069 | 0.10 | -20.62 | 1.69 | -30.96 |
| 25.94 | 0.085 | 0.03 | -22.42 | 1.68 | -26.68 |
| 30.19 | 0.102 | 0.02 | -22.92 | 2.28 | -25.58 |
| 34.44 | 0.121 | 0.09 | -19.79 | 2.41 | -29.46 |
| 38.69 | 0.142 | 0.03 | -22.15 | 1.82 | -30.25 |
| 42.93 | 0.164 | 0.14 | -21.89 | 2.02 | -29.50 |
| 47.18 | 0.187 | 0.08 | -20.69 | 1.67 | -25.77 |
| 51.43 | 0.212 | 0.08 | -19.66 |  |  |
| 55.68 | 0.238 | 0.04 | -22.00 |  |  |
| 59.93 | 0.266 | 0.08 | -22.81 | 1.21 | -30.20 |
| 64.17 | 0.295 | 0.03 | -24.00 | 1.96 | -30.52 |
| 68.42 | 0.326 | 0.11 | -19.39 | 2.10 | -30.75 |
| 72.67 | 0.358 | 0.08 | -19.09 | 1.67 | -30.58 |
| 76.92 | 0.391 | 0.04 | -23.92 | 2.10 | -27.88 |
| 81.16 | 0.426 | 0.09 | -20.61 | 1.77 | -30.45 |
| 85.41 | 0.463 | 0.05 | -22.21 | 2.11 | -31.68 |
| 89.66 | 0.500 | 0.02 | -21.44 | 0.90 | -30.68 |
| 93.91 | 0.540 | 0.04 | -21.86 | 1.63 | -30.97 |
| 98.15 | 0.580 | 0.03 | -21.24 | 2.55 | -33.15 |
| 106.65 | 0.666 | 0.03 | -22.30 | 1.11 | -31.10 |
| 110.90 | 0.711 | 0.09 | -21.84 | 1.23 | -30.71 |
| 115.14 | 0.758 | 0.09 | -18.82 | 1.41 | -30.95 |
| 119.39 | 0.806 | 0.15 | -21.36 | 1.44 | -31.54 |
| 123.64 | 0.855 | 0.03 | -21.94 | 1.78 | -28.94 |
| 127.89 | 0.906 | 0.17 | -19.43 | 1.58 | -27.33 |
| 132.13 | 0.958 | 0.07 | -19.24 | 1.06 | -30.98 |
| 136.38 | 1.012 | 0.05 | -22.19 | 2.18 | -30.56 |
| 140.63 | 1.067 | 0.04 | -22.06 | 2.80 | -32.89 |
| 144.88 | 1.123 | 0.07 | -19.07 | 1.91 | -31.27 |
| 149.12 | 1.181 | 0.07 | -20.09 | 2.27 | -30.62 |
| 153.37 | 1.241 | 0.08 | -22.78 | 1.61 | -30.36 |
| 157.62 | 1.302 | 0.10 | -18.82 | 1.22 | -30.00 |
| 161.87 | 1.364 | 0.06 | -18.35 | 1.05 | -30.83 |
| 166.12 | 1.428 | 0.09 | -22.79 | 1.46 | -29.67 |
| 170.36 | 1.432 | 0.02 | -20.58 | 1.45 | -30.51 |
| 174.61 | 1.532 | 0.07 | -19.17 | 0.98 | -30.32 |
| 178.86 | 1.633 | 0.02 | -20.67 | 0.98 | -30.54 |
| 183.11 | 1.735 | 0.06 | -17.50 | 1.64 | -30.48 |
| 187.35 | 1.838 | 0.08 | -18.39 | 1.77 | -30.87 |
| 191.60 | 1.941 | 0.03 | -22.10 | 1.35 | -30.67 |
| 195.85 | 2.045 | 0.08 | -20.42 | 1.32 | -31.11 |
| 200.10 | 2.149 | 0.05 | -17.19 | 1.97 | -30.85 |
| 204.34 | 2.254 | 0.09 | -18.63 | 1.50 | -30.90 |
| 208.59 | 2.360 | 0.07 | -20.21 |  |  |
| 212.84 | 2.467 | 0.08 | -19.20 | 1.35 | -30.53 |
| 217.09 | 2.575 | 0.07 | -22.82 | 1.91 | -30.96 |
| 221.33 | 2.683 | 0.03 | -18.92 | 1.21 | -30.13 |
| 225.58 | 2.792 | 0.05 | -22.19 | 1.05 | -31.21 |
| 229.83 | 2.901 | 0.01 | -19.14 | 1.50 | -29.94 |
| 234.08 | 3.012 | 0.06 | -19.49 | 1.37 | -30.68 |
| 251.07 | 3.460 | 0.08 | -19.87 | 0.80 | -30.38 |
| 255.31 | 3.574 | 0.07 | -22.70 | 1.97 | -30.11 |
| 259.56 | 3.689 | 0.02 | -19.34 | 1.38 | -30.56 |
| 263.81 | 3.804 | 0.06 | -22.87 | 1.56 | -29.75 |
| 268.06 | 3.920 | 0.10 | -19.75 | 1.32 | -30.57 |
| 272.31 | 4.037 | 0.07 | -23.57 | 1.47 | -30.68 |
| 276.55 | 4.155 | 0.04 | -22.93 | 1.35 | -30.23 |
| 280.80 | 4.273 | 0.04 | -18.05 | 1.25 | -30.65 |
| 285.05 | 4.392 | 0.05 | -17.94 | 0.63 | -30.28 |
| 289.30 | 4.512 | 0.07 | -22.33 | 1.08 | -30.82 |
| 293.54 | 4.632 | 0.03 | -18.24 | 1.45 | -30.78 |
| 297.79 | 4.753 | 0.04 | -21.10 | 0.77 | -30.99 |
| 302.04 | 4.875 | 0.03 | -22.63 | 1.28 | -30.13 |
| 306.29 | 4.998 | 0.06 | -21.13 | 1.08 | -31.07 |
| 310.53 | 5.121 | 0.07 | -22.11 | 1.87 | -29.58 |
| 314.78 | 5.245 | 0.06 | -18.03 |  | -30.58 |
| 323.28 | 5.496 | 0.08 | -18.60 | 1.07 | -29.55 |
| 327.52 | 5.622 | 0.04 | -19.75 | 1.56 | -29.54 |
| 340.27 | 6.005 | 0.06 | -24.47 | 1.62 | -29.70 |
| 344.51 | 6.134 | 0.02 | -20.72 | 1.63 | -30.41 |
| 348.76 | 6.264 | 0.03 | -22.55 | 1.23 | -30.64 |
| 353.01 | 6.394 | 0.09 | -21.53 | 1.27 | -29.24 |
| 357.26 | 6.526 | 0.07 | -18.69 | 1.47 | -29.09 |
| 361.50 | 6.658 | 0.03 | -22.58 | 1.38 | -31.26 |
| 365.75 | 6.790 | 0.03 | -21.66 |  | -30.07 |
| 370.00 | 6.924 | 0.06 | -18.92 | 1.90 | -30.66 |
| 374.25 | 7.058 | 0.06 | -19.40 | 1.54 | -30.25 |
| 378.50 | 7.193 | 0.04 |  | 1.34 | -30.61 |
| 382.74 | 7.328 | 0.04 | -22.93 | 1.27 | -30.82 |
| 386.99 | 7.465 | 0.02 | -21.39 | 1.18 | -30.07 |
| 391.24 | 7.602 | 0.07 | -25.00 | 0.99 | -30.03 |
| 395.49 | 7.739 | 0.06 | -20.49 | 0.67 | -28.24 |
| 399.73 | 7.878 | 0.07 | -21.96 | 1.16 | -29.40 |
| 403.98 | 8.017 | 0.03 | -22.49 | 1.38 | -29.80 |
| 408.23 | 8.157 | 0.09 |  |  |  |
| 412.48 | 8.298 | 0.06 |  |  |  |
| 416.72 | 8.439 | 0.07 | -19.84 | 0.99 | -30.12 |
| 420.97 | 8.700 | 0.07 | -23.55 |  |  |
| 425.22 | 9.167 | 0.04 | -20.07 | 1.11 | -30.39 |
| 429.47 | 9.416 | 0.06 | -19.52 | 0.93 | -30.34 |
| 433.71 | 9.662 | 0.08 |  | 1.10 | -30.18 |
| 437.96 | 9.907 | 0.03 | -22.02 | 1.29 | -30.32 |
| 442.21 | 10.149 | 0.06 | -22.04 | 0.85 | -29.83 |
| 450.70 | 10.626 | 0.07 | -18.95 | 1.40 | -29.61 |
| 454.95 | 10.862 | 0.06 | -20.43 | 1.94 | -30.18 |
| 459.20 | 11.095 | 0.07 | -23.15 | 1.82 | -29.92 |
| 463.45 | 11.327 | 0.02 | -22.42 | 1.73 | -30.54 |
| 467.69 | 11.556 | 0.03 | -23.44 | 0.74 | -28.95 |
| 471.94 | 11.783 | 0.07 | -19.90 | 1.48 | -29.06 |
| 476.19 | 12.007 | 0.06 | -21.12 | 0.60 | -29.52 |
| 480.44 | 12.230 | 0.06 | -20.62 | 0.77 | -28.72 |
| 484.69 | 12.450 | 0.05 | -21.81 | 0.93 | -28.37 |
| 488.93 | 12.669 | 0.04 | -24.28 | 0.98 | -30.88 |
| 493.18 | 12.885 | 0.06 | -20.99 | 0.40 |  |
| 497.43 | 13.099 | 0.06 | -22.05 | 0.54 | -29.66 |
| 501.68 | 13.310 | 0.05 | -20.54 | 0.67 |  |
| 505.92 | 13.520 | 0.05 | -20.63 | 0.65 | -30.18 |
| 510.17 | 13.727 | 0.08 | -18.02 |  |  |
| 514.42 | 13.933 | 0.06 | -19.12 | 1.77 | -29.41 |
| 518.67 | 14.136 | 0.02 | -22.55 | 0.85 | -28.72 |
| 522.91 | 14.337 | 0.05 | -21.49 | 1.23 | -28.81 |
| 527.16 | 14.535 | 0.06 | -21.43 | 1.12 | -29.27 |
| 531.41 | 14.732 | 0.03 | -24.39 | 0.71 | -29.79 |
| 535.66 | 14.926 | 0.03 | -23.17 | 0.74 | -29.74 |
| 539.90 | 15.119 | 0.06 | -20.80 | 1.22 | -28.94 |
| 544.15 | 15.309 | 0.05 | -21.14 | 1.73 | -29.23 |
| 548.40 | 15.497 | 0.05 | -21.19 | 1.05 | -29.77 |
| 552.65 | 15.683 | 0.06 | -24.72 | 1.23 | -29.89 |
| 556.89 | 15.866 | 0.02 | -23.01 | 0.71 | -29.82 |
| 561.14 | 16.048 | 0.04 | -22.42 | 0.92 | -29.36 |
| 565.39 | 16.227 | 0.03 | -21.30 | 1.06 | -28.95 |
| 569.64 | 16.404 | 0.06 | -21.96 | 0.89 | -29.11 |
| 573.88 | 16.579 | 0.05 | -21.86 | 0.84 | -29.85 |
| 578.13 | 16.752 | 0.04 | -23.01 | 0.80 | -28.66 |
| 582.38 | 16.922 | 0.04 | -22.97 | 0.79 | -29.83 |
| 586.63 | 17.091 | 0.05 | -22.04 | 0.92 | -29.69 |
| 590.88 | 17.257 | 0.05 | -21.94 | 0.76 | -30.59 |
| 595.12 | 17.421 | 0.04 | -21.29 | 0.84 | -29.21 |
| 599.37 | 17.583 | 0.05 | -21.22 | 1.04 | -30.17 |
| 603.62 | 17.743 | 0.05 | -21.54 | 0.77 | -29.86 |
| 607.87 | 17.901 | 0.05 | -21.59 | 1.21 | -29.25 |
| 612.11 | 18.056 | 0.03 | -23.15 | 1.10 | -29.45 |
| 616.36 | 18.209 |  | -23.17 | 1.49 | -29.08 |
| 620.61 | 18.361 | 0.08 | -20.43 | 1.36 | -29.22 |
| 624.86 | 18.509 | 0.05 | -21.59 | 1.53 | -29.66 |
| 629.10 | 18.656 | 0.05 | -21.66 | 1.06 | -30.43 |
| 633.35 | 18.801 | 0.06 | -20.93 | 0.94 | -29.78 |
| 637.60 | 18.943 | 0.05 | -19.78 | 1.60 | -29.76 |
| 641.85 | 19.084 | 0.07 | -20.98 | 1.40 | -29.25 |

**References:**

1. Shipboard Scientific Party. Site 1146. Proceedings of the Ocean Drilling Program, Part A: Initial Reports, 184 (2000).

2. Deines P. The isotopic composition of reduced organic carbon in Handbook of environmental isotope Geochemistry I, the terrestrial environment (Fritz, P. & Fontes, J. C.), 329-406 (1980).

3. Cerling, T. et al. Global vegetation change through the Miocene /Pliocene boundary. *Nature***389,** 153-158 (1997).

4. Kohn, M. J. Carbon isotope composition of terrestrial C_3_ plants as indicators of (paleo) ecology and (paleo) climate. *Proceedings of the National Academy of Sciences USA***107**, 19691-19695 (2010).

5. Liu, W. et al. Carbon isotopic composition of modern soil and paleosol as a response to vegetation change on the Chinese Loess Plateau. S*ci China Ser D-Earth Sci*48, 93-99 (2002).

6. Zheng, S. & Shangguan, Z. Spatial patterns of foliar stable isotope composition of C_3_ plant species in the Loess Plateau of China. *Ecological Research***22**, 242-353 (2007).

7. Bird, M., & Gröcke, D. Determination of the abundance and carbon isotope composition of elemental carbon in sediments. *Geochimica et Cosmochimica Acta***61**, 3413-3423 (1997).

8. Liu, L., Yang, S., Cui, L. & Hao, Z. Stable carbon isotopic composition of black carbon in surface soil as a proxy for reconstructing vegetation on the Chinese Loess Plateau. *Palaeogeography, Palaeoclimatology, Palaeoecology***388**, 109-114 (2013).

9. Bird, M., & Ascough, P. Isotopes in pyrogenic carbon: A review. *Organic Geochemistry***42**, 1529-1539 (2012).

10. Diefendorf, A. F., Mueller, Scott, K. E., Wang, L., Koch, P. L., & Freeman, K. H. Global patterns in leaf ^13^C discrimination and implications for studies of past and future climate. *Proceedings of the National Academy of Sciences USA***107,** 5738–5743 (2010).

11. Bi, X., Sheng, G., Liu, X., Li, C. & Fu, J. Molecular and carbon and hydrogen isotopic composition of n-alkanes in plant waxes. *Organic Geochemistry***36**, 1405-1417 (2005).

12. Tipple, B. & Pagani, M. A 35 Myr North American leaf-wax compound-specific carbon and hydrogen isotope record: implications for C4 grasslands and hydrologic cycle dynamics. *Earth and Planetary Science Letters***299**, 250-262 (2010).

13. Tipple, B., Meyers, S. & Pagani, M. Carbon isotope ratio of Cenozoic CO2: A comparative evaluation of available geochemical proxies. *Paleoceanography***25**, PA3202 (2010).

14. Schubert, B. & Jahren, H. The effect of atmospheric CO_2_ concentration on carbon isotope fractionation in C_3_ land plants. *Geochimica et Cosmochimica Acta***96**, 29-43 (2012).

15. Schubert, B. & Jahren, H. Global increase in plant carbon isotope fractionation following the Last Glacial Maximum caused by increase in atmospheric pCO_2_. *Geology***43**, 435-438 (2015).

16. Beerling, D. & Royer, D. Convergent Cenozoic CO_2_ history. *Nature Geoscience***4**, 418-420 (2011).

17. Pearson, P. & Palmer, M. Atmospheric carbon dioxide concentrations over the past 60 million years. *Nature***406**, 695-699 (2000).

18. Tripati, A., Roberts, C., Eagle, R. Coupling of CO2 and ice sheet stability over major climate transitions of the last 20 million years. *Science***326**, 1394-1397 (2009).

19. Gustafsson, O. et al. Evaluation of a protocol for the quantification of black carbon in sediments. *Global Biogeochemical Cycles***15**, 881-890 (2001).

20. Thevenon, F. et al. Combining charcoal and elemental black carbon analysis in sedimentary archives: Implications for past fire regimes, the pyrogenic carbon cycle, and the human–climate interactions. *Global & Planetary Change***72,** 381-389 (2010).

21. Wu, D. et al. Black carbon over the South China Sea and in various continental locations in South China. *Atmos. Chem. Phys*.**13**, 12257-12270 (2013).

23. Lee, Y. et al. An integrated approach to identify the biomass burning sources contributing to black carbon epidodes in Hong Kong. *Atmospheric Environment***80**, 478-487 (2013).

24. Lin, N. et al. An overview of regional experiments on biomass burning aerosos and related pollutants. *Atmopheric Environment***78**, 1-19 (2013).

25. Chuang, M. et al. Aerosol chemical properties and related pollutants measured in Dongsha Island in the northern South China Sea during –SEAS/Dongsha Experiment. *Atmospheric Environment***78**, 82-92 (2013).

26. Wang, S. et al. First detailed observations of long-range transported dust over the northern South China Sea. *Atmospheric Environment***45**, 4804-4808 (2011).

27. Wang, S. et al. Origin, transport, and vertical distribution of atmospheric pollutants over the northern South China Sea during the 7-SEAS/Dongsha Experiment. *Atmospheric Environment***78**, 124-133 (2013).

28. Wang, L. et al. East Asian monsoon climate during the Late Pleistocene: high-resolution sediment records from the South China Sea. *Marine Geology***156**, 145-284 (1999).

29. Sun, X. & Li, X. A pollen record of the last 37 ka in deep sea core 17940 from the northern slope of the South China Sea. *Marine Geology***156,** 227-244 (1999).

30. Pelejero, C. Terrigenous n-alkane input in the South China Sea: High-resolution records and surface sediments. *Chemical Geology***200**, 89-103 (2003).

31. Zhao, Y. et al. Non-polar organic compounds in marine aerosols over the northern South China Sea: Influence of continental outflow. *Chemosphere***153**, 332-339 (2016).

32. Ma, P., Gattiker, J., Liu, X. & Rasch, P. A novel approach for determining source-receptor relationships in model simulations: a case study of black carbon transport in northern hemisphere winter. *Environmental Research Letters***8**, 024042-24049 (2013).

32. Zhou, B. et al. Late Pliocene-Pleistocene expansion of C_4_ vegetation in semi-arid East Asia linked to increased burning. *Geology***42**, 1067-1070 (2014).

33. Jia, G., Peng, P., Zhao, Q. & Jian, Z. Changes in terrestrial ecosystem since 30 Ma in East Asia: stable isotope evidence from black carbon in the South China Sea. *Geology***31**, 1093-1096 (2003).

34. Wan, S., Li, A., Clift, P. & Stuut, J. Development of the East Asian monsoon: Mineralogical and sedimentologic records in the northern South China Sea since 20 Ma. *Palaeogeography, Palaeoclimatology, Palaeoecology***254**, 561-582 (2007).

35. Clift, P., Wan, S. & Bluaxtajn, J. Reconstructing chemical weathering, physical erosion and monsoon intensity since 25 Ma in the northern South China Sea: A review of competing proxies. *Earth-Science Reviews***130**, 86-102 (2014).
